# Bioprospecting Plant Growth-Promoting Rhizobacteria That Mitigate Drought Stress in Grasses

**DOI:** 10.3389/fmicb.2019.02106

**Published:** 2019-09-10

**Authors:** Michael D. Jochum, Kelsey L. McWilliams, Eli J. Borrego, Mike V. Kolomiets, Genhua Niu, Elizabeth A. Pierson, Young-Ki Jo

**Affiliations:** ^1^Department of Plant Pathology and Microbiology, Texas A&M University, College Station, TX, United States; ^2^Thomas H. Gosnell School of Life Sciences, Rochester Institute of Technology, Rochester, NY, United States; ^3^Texas A&M AgriLife Research and Extension Center, El Paso, TX, United States; ^4^Department of Horticultural Sciences, Texas A&M University, College Station, TX, United States

**Keywords:** PGPR, drought, bioprospecting, plant, growth-promoting, rhizobacteria, wheat

## Abstract

This study reports the application of a novel bioprospecting procedure designed to screen plant growth-promoting rhizobacteria (PGPR) capable of rapidly colonizing the rhizosphere and mitigating drought stress in multiple hosts. Two PGPR strains were isolated by this bioprospecting screening assay and identified as *Bacillus* sp. (12D6) and *Enterobacter* sp. (16i). When inoculated into the rhizospheres of wheat (*Triticum aestivum*) and maize (*Zea mays*) seedlings, these PGPR resulted in delays in the onset of plant drought symptoms. The plant phenotype responding to drought stress was associated with alterations in root system architecture. In wheat, both PGPR isolates significantly increased root branching, and *Bacillus* sp. (12D6), in particular, increased root length, when compared to the control. In maize, both PGPR isolates significantly increased root length, root surface area and number of tips when compared to the control. *Enterobacter* sp. (16i) exhibited greater effects in root length, diameter and branching when compared to *Bacillus* sp. (12D6) or the control. *In vitro* phytohormone profiling of PGPR pellets and filtrates using LC/MS demonstrated that both PGPR strains produced and excreted indole-3-acetic acid (IAA) and salicylic acid (SA) when compared to other phytohormones. The positive effects of PGPR inoculation occurred concurrently with the onset of water deficit, demonstrating the potential of the PGPR identified from this bioprospecting pipeline for use in crop production systems under drought stress.

## Introduction

Drought is a major abiotic stress threatening agricultural production worldwide. In the last 40 years, drought stress has reduced yields in cereals by as much as 10% (Lesk et al., 2016) and is forecasted to affect production on over 50% of the arable land by 2050 (Vinocur and Altman, 2005). In order to address this global challenge in agriculture, research has focused on improving germplasm and developing crop management practices to increase water use efficiency (Passioura, 2007; Ngumbi and Kloepper, 2016). However, recent attention has turned to the application of beneficial microorganisms that mediate drought tolerance and improve plant water-use efficiency and these efforts have been augmented due to technological advances in next generation sequencing and microbiomics (Dimkpa et al., 2009; Marulanda et al., 2009; Yang et al., 2009; Ngumbi and Kloepper, 2016; Vurukonda et al., 2016).

The application of plant growth-promoting rhizobacteria (PGPR) is considered a sustainable synergistic biological approach to cope with water deficiency in crop production. PGPR readily colonize the root rhizosphere and establish both free-living and intimate associations with host plants. Often, these interactions lead to enhancement of crop productivity and mitigation of biotic and abiotic stresses through a variety of mechanisms (Mayak et al., 2004; Berg, 2009; Dimkpa et al., 2009; Liu et al., 2013; Mendes et al., 2013; Vacheron et al., 2013; Porcel et al., 2014; Gontia-Mishra et al., 2016; Ngumbi and Kloepper, 2016; Vurukonda et al., 2016; Barnawal et al., 2017; Forni et al., 2017). PGPR may play critical roles as suppressors of plant disease, biofertilizers, alleviators of abiotic stress and remediators of toxins from the soil (Mayak et al., 2004; Naveed et al., 2014; Timmusk et al., 2014). Mechanisms associated with PGPR-derived drought tolerance include alterations in host root system architecture, osmoregulation, management of oxidative stress via the biosynthesis and metabolism of phytohormones or the production of antioxidants for scavenging reactive oxygen species (ROS), the production of large chain extracellular polysaccharide (EPS) that may serve as humectant, and transcriptional regulation of host stress response genes (Dimkpa et al., 2009; Liu et al., 2013; Vacheron et al., 2013; Osakabe et al., 2014; Timmusk et al., 2014; Gontia-Mishra et al., 2016; Ngumbi and Kloepper, 2016; Vurukonda et al., 2016; Barnawal et al., 2017; Forni et al., 2017).

The objective of this study was to design and implement a bioprospecting screen to isolate PGPR capable of rapidly colonizing seedling rhizospheres and mediating drought stress in multiple cereal hosts. For this purpose, the screening method was developed that emphasized the following: (a) A selection of a likely source containing PGPR, (b) A pre-screening process focused on desired plant phenotypes, and (c) A final screening process focused on candidates likely to provide desired outcomes under practical production practices on both wheat and maize.

The original source of PGPR were the rhizospheres of perennial grasses collected from El Paso, TX, where the semi-arid environment provides a strong selective pressure for survival under nearly constant water deficit. The rationale for choosing the starting material was that perennial grasses growing vigorously under pervasive water stress conditions were likely to foster a microbiome capable of mitigating drought stress. The pre-screening process focused on the desired host phenotype, rather than bacterial phenotypes. The host phenotype used for screening was the delayed of onset of drought stress symptoms in seedlings, since seedling establishment is often the most vulnerable stage and may have large impacts on crop stand and yield (Pessarakli, 1999). The final selection process focused on the identification of PGPR that are most likely to have applications in existing commercial production systems. Given current limitations in “seed space” for new growth stimulating products combined with the difficulties in reliable formulation of application-friendly seed treatments, the focus of this study was on identifying isolates that could be applied as needed prior to the onset of water stress conditions. The screening protocol was designed to specifically select isolates that could rapidly colonize and provide benefits to the host, e.g., if inoculated at the onset of water deficit conditions. In this manner, this screen provides the unique ability to select strains that can be added as needed, as compared to current seed coating applications. Isolated candidate PGPR strains demonstrating robust effectiveness were validated on two different grass hosts, wheat (*T. aestivum*) and maize (*Z. mays*).

## Materials and Methods

### Rhizobacteria Sampling and Screening

Twenty-five bermudagrass (*Cynodon* spp.) thatch core samples (10 cm diameter and 15 cm depth) were collected in the summer of 2015 and 2016 in El Paso, TX, United States. Sampling sites included medians, parks, roadsides and ranches. Intact core samples were immediately shipped upon removal under ambient temperatures to the lab in College Station, TX. Each sample core was then subdivided into 5 cm diameter cores, transferred to a round plastic pot (10 cm diameter, 12 cm height) with holes in the bottom, filled-in with autoclaved potting mix (Metro-Mix 900, Sun Gro Horticulture, Agawam, MA, United States), and grown in a greenhouse for 14 days. Grasses were exposed to three different levels of watering: non-stressed (watering up to the field capacity every other day), moderate stress (watering once a week), and severe stress (no watering). The onset of drought symptoms was daily monitored and recorded based on phenotype: leaf wilting, curling, tip burning, and plant lodging. The five cores containing plants for which drought symptoms were most delayed under both the moderate and severe watering regimes were used for the next step: bacterial isolation and preservation for screening trials. By conducting this pre-screening of grass samples in a controlled setting, we mitigate the possibility of sampling habitats of compensation that demonstrated drought resistant phenotypes due to source-sink effects (Leibold et al., 2004).

Rhizosphere samples for bacterial isolation were obtained from one gram of root tissue, excised from the grasses in each of the selected cores. Root tissue samples were first washed in sterile dH_2_O to remove detritus and non-root adherent soil, suspended in 10 ml of 0.1 M phosphate buffered saline (1 min), and macerated using a drill homogenizer (115V Bio-Gen PRO200 homogenizer unit, 5 × 75 mm generator probe). PBS suspensions were serially diluted and plated on Luria-Bertani (LB) agar amended with 5 mg L^–1^ cycloheximide and 10% sorbitol (Kavamura et al., 2013). Plates were maintained 25°C and inspected daily for bacterial growth. Morphologically distinct colonies were re-isolated to obtain axenic cultures and then grown separately overnight in LB broth (25°C, 120 rpm agitation) and stored in 40% glycerol at −80°C.

### PGPR Screening

Wheat (*T. aestivum* subsp. *aestivum* cultivar TAM111) and maize (*Z. mays* cultivar B73) seeds were surface sterilized in 10% NaOCl for 10 min, followed by 10 subsequent rinses in sterile dH_2_O. Seeds were germinated on sterile filter paper 24 h at 37°C for wheat and 25°C for maize. Germinated seeds were planted separately in pots (10 cm diameter, 12 cm height) with holes containing 400 g sterilized Metro-Mix 900. Seedlings were watered to field capacity every day, determined by water leaching through the bottom of the pot, and cultivated in a growth chamber for 7 days (30°C, using fluorescent bulbs emitting 300 μmol m^–2^ s^–1^, 12:12 h light and dark cycle). Plants were inoculated 7 days post germination with test strains, followed by withholding water for the next 7 days. For the bacterial inoculum, overnight cultures were grown in LB at 25°C, collected via centrifugation (2,500×*g*, 5 min) and re-suspended in an equal volume of 0.1 M PBS. 80 μl of resuspended inoculum was applied to the soil at the base of each seedling. Inoculation with 0.1 M PBS was used as a no-inoculum control. For PGPR isolates that showed positive activity, in subsequent trials, inoculum densities were regulated to insure populations of approximately 10^7^ colony-forming unit (CFU) ml^–1^ via optical density (600 nm) measurements. Growth curves comparing colony counts and optical density were used to determine the optical densities that provided the desired population densities.

### Drought Tolerance Phenotyping

At the end of the 7-day water stress treatment (14 days post planting), inoculated and non-inoculated plants were examined for drought symptoms such as wilting, leaf curling and marginal leaf necrosis. Plants were then removed from the soil, with special care to preserve the intact root system. Roots were washed to remove soil and detritus via spraying with dH_2_O against a 0.5 mm mesh sieve. Harvested root and shoot tissues were saturated with dH_2_O via storage in wet germination paper at 4°C overnight, in preparation for downstream analysis (Himmelbauer et al., 2004). Washed roots were separated from above ground tissue, submerged in dH_2_O and spread out to prevent overlap in a root positioning tray (20 × 30 cm) with three roots per tray. Roots were scanned using a flatbed scanner (EPSON, Perfection V-750). Root image data obtained by scanning were analyzed using WinRHIZO Arabidopsis 2017a (WinRHIZO, RRID:SCR_017120), generating estimates of total root length, root surface area, average root diameter, number of root tips, and root branching as previously described (Arsenault et al., 1995; Himmelbauer et al., 2004). For plants that exhibited delayed drought stress symptoms relative to control plants, bacterial population sizes were determined via serial dilution plating. In all experiments, root population sizes were 10^6^–10^7^ CFU g^–1^ of rhizosphere, defined as root and root adherent soil. Bacteria were re-isolated from root rhizosphere on LB amended with cycloheximide and stored as before.

The experiment for evaluating drought tolerance phenotypes by PGPR was conducted in a completely randomized block design with five replications (plants). The experiment was repeated once. Plant phenotype data from WinRHIZO and LC-MS results were analyzed using an analysis of variance (ANOVA) (Statistical Analysis System, RRID:SCR_008567). Pairwise comparisons between the treatments were conducted using Fischer’s least significant difference (LSD) test at *P* = 0.05. Root scans and statistical analysis scripts can be found at the https://github.tamu.edu/jochum00/04_16_2019_SAS.

### Isolate Sequencing

For bacterial strains of interest, taxonomic information was obtained via sequencing of the 16S and 23S ribosomal RNA subunit and ITS regions (Stackebrandt and Goebel, 1994; Dinesh et al., 2015). Genomic DNA from each strain was extracted using the CTAB protocol (William et al., 2012). Polymerase chain reaction (PCR) was used to amplify the target region with the following primers: 16S region forward 8F/pA (5′-GAGTTTGATCCTGGCTCAG-3′) and 23s reverse p23SR01 (5′-GCTGCTTCTAAGCCAAC-3′) (Stackebrandt and Goebel, 1994; Dinesh et al., 2015). PCR was performed in a thermocycler (Applied Biosystems Thermocycler 2720) with the following reaction conditions: 1 min 95°C; 35 cycles of 1 min 95°C, 1 min 52.7°C, and 1.5 min 72°C; 1 cycle 10 min 72°C; maintain at 4°C until retrieval. PCR amplicons were gel purified using the Wizard SV Gel and PCR Clean-Up System (Promega, RRID:SCR_006724), and sequenced (Eton Bioscience, RRID:SCR_003533) with the aforementioned PCR primers and sequencing primers 1542R/pHr (5′-TGCGGCTGGATCACCTCCTT-3′) and 1542R/pH (5′-AAGGAGGTGATCCAGCCGCA-3′). The reads were aligned using MAFFT algorithm in Benchling (Benchling, RRID:SCR_013955). Consensus alignments were taxonomically identified at the genus level via NCBI nucleotide Basic Local Alignment Search Tool (BLASTN, RRID:SCR_001598).

### Phytohormone Profiling

Ten milliliters of LB overnight cultures from each strain were pelleted via centrifugation at 10,000 rpm for 10 min. Supernatants were decanted into a Nalgene^®^ Rapid-Flow^TM^ sterilization filter unit containing a 0.2 μm nitrate cellulose membrane and filtered via vacuum filtration. Pellet and filtrate samples were lyophilized for 24 h., followed by resuspension in 500 μl extraction buffer consisting of n-propanol, H_2_O and HCl (2:1:0.002 by volume) spiked with 500 nM of following deuterated internal standards: d-ABA ([^2^H_6_] (+)-*cis*,*trans-*abscisic acid; Olchemlm cat# 0342721), d-ACC (1-Aminocyclopropane-2,2,3,3-d4-carboxylic acid; Sigma cat#736260), d-*trans-*Cinnamic acid (d7- cinnamic acid; Sigma cat#513954), d-IAA([2H5] indole-3-acetic acid; Olchemlm cat# 0311 531), d-JA (2,4,4-d3; acetyl-2,2-d2 jasmonic acid; CDN Isotopes cat# D-6936), and d-SA (d6- salicylic acid; Sigma cat#616796). Following resuspension, we conducted phase separation via the addition of dicholormethane (CH_2_Cl_2_) for 30 min at 4°C, followed by centrifugation at 14,000 rpm for 10 min. The organic phase was removed, evaporated under N_2_ gas in a glass vial, followed by re-solubilization in 150 μl methanol precipitation and incubated overnight in −20°C. Samples were then centrifuged at 14,000 rpm for 5 min. After centrifugation, 10 μl of supernatant from each sample were injected into a C18 analytical column for liquid chromatography analyte separation, followed by detection via triple quadruple mass spectrometry. Samples were quantified for phytohormones and oxylipins via comparison against the internal deuterated standards as previously described (Stumpe et al., 2005; Strauch et al., 2015).

## Results

**FIGURE 1:**
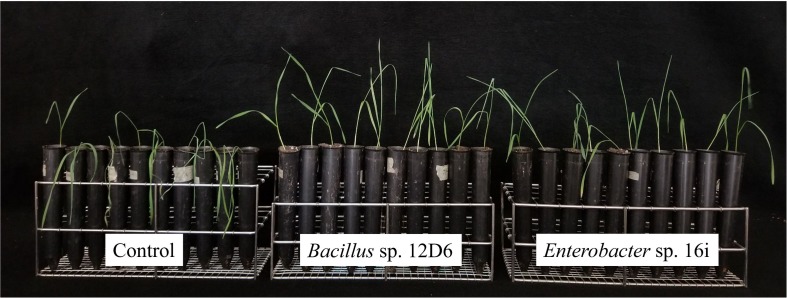
Wheat seedlings treated with plant growth-promoting rhizobacteria (PGPR). *Bacillus* sp. 12D6 **(middle)** and *Enterobacter* sp. 16i **(right)** demonstrated the delayed onset of drought symptoms versus control **(left)** in wheat seedlings after exposure to 7 days of continuous water deficit.

**FIGURE 2:**
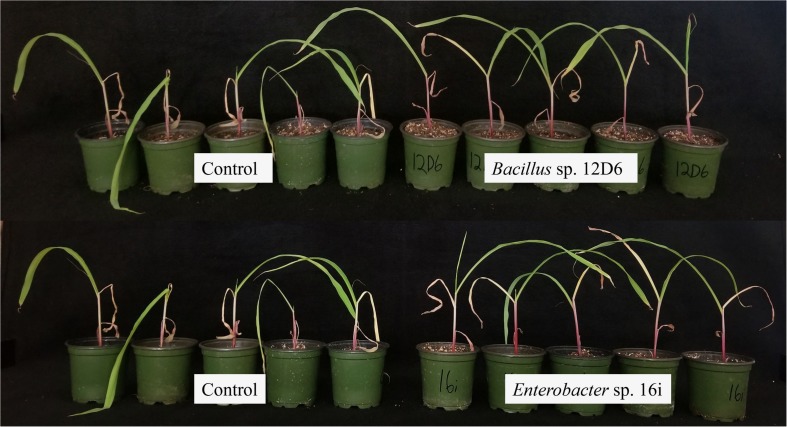
Maize seedlings treated with plant growth-promoting rhizobacteria (PGPR). *Bacillus* sp. 12D6 **(top right)** and *Enterobacter* sp. 16i **(bottom right)** demonstrated the delayed onset of drought symptoms in maize seedlings versus control **(top left** and **bottom left)** after exposure to 7 days of continuous water deficit.

Out of 200 isolates tested, soil inoculation by two PGPR strains, 12D6 and 16i, significantly alleviated drought stress symptoms in both wheat (Figure 1) and maize (Figure 2) seedlings. Qualitative assessment of plant performance across replicate experiments suggested strain 12D6 was somewhat more effective in mediating a delay in the onset of drought symptoms in wheat, whereas strain 16i was more effective in mediating this effect in maize. Results from the NCBI BLASTN query based on rRNA sequence identified strain 12D6 (accession no. MH678658 and MH683042) as *Bacillus* sp. (ident = 99%) and 16i (accession no. MH678659 and MH683043) as *Enterobacter* sp. (ident = 99%).

Results from a two-way ANOVA (host × bacterial treatment) revealed that given the larger size of the maize root system compared to the wheat root system, all maize root system dependent variables were statistically larger than those of wheat (*P* < 0.0001). Consequently, the ANOVA was performed separately for each host (Table 1).

**TABLE 1:** Analysis of variance (ANOVA) for the effect of plant growth-promoting rhizobacteria (PGPR) treatment on wheat and maize root systems following a 7-day water deficit.

**Dependent variable**	**Wheat**				**Maize**			
	***df***	**Mean squared**	***F***	***P***	***df***	**Mean squared**	***F***	***P***
Root length	2	4512.80756	3.13	0.0599	2	26904.8926	13.89	< 0.0001
Root surface area	2	23.16365397	1.93	0.1653	2	147.012501	4.48	0.0198
Average diameter	2	0.00067100	1.29	0.2929	2	0.00723170	8.82	0.0010
Root tips	2	69630.700	1.42	0.2596	2	207948.394	5.02	0.0132
Root branching	2	110906.8000	4.91	0.0152	2	512832.212	8.72	0.0010

**FIGURE 3:**
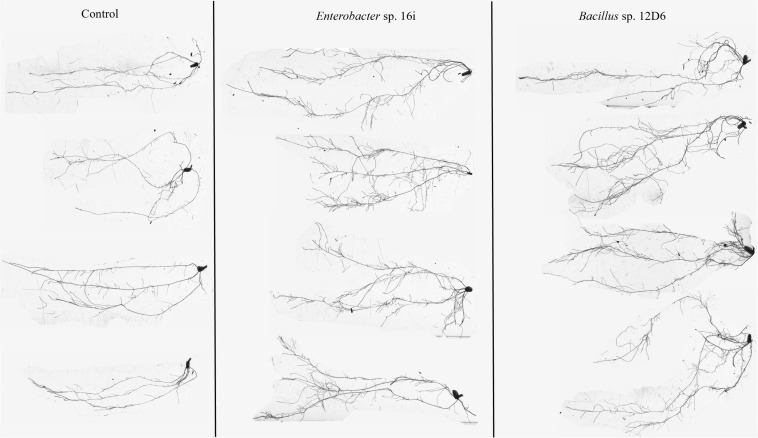
Root system architecture in wheat seedlings treated with the control **(left)**, *Enterobacter* sp. 16i **(center)** and *Bacillus* sp. 12D6 **(right)** after exposure to 7 days of continuous water deficit.

In wheat, the root systems of seedlings (Figure 3) treated with either bacterial inoculum were more branched than those of the non-inoculated seedlings. Treatment of seedlings with *Bacillus* sp. (12D6) contributed to greater total root length compared to the control treatment (Table 2).

**TABLE 2:** Pairwise comparisons using Fischer’s LSD test (*n* = 10) of wheat and maize root system architecture with and without plant growth-promoting rhizobacteria (PGPR) inoculation, analyzed using WinRHIZO software.

**Host plant**	**Treatment**	**Root length (cm)**	**Root surface area (cm^2^)**	**Average diameter (mm)**	**Number of root tips**	**Number of root branching**
Wheat	*Bacillus* sp. 12D6	165.40A	11.88	0.248	676.2	604.6A
	*Enterobacter* sp. 16i	161.49*AB*	12.08	0.236	628.3	544.8A
	Control	126.81B	9.35	0.233	513.8	399.8B
Maize	*Bacillus* sp. 12D6	323.94B	40.49A	0.399B	1149.8A	1299.6B
	*Enterobacter* sp. 16i	370.16A	42.55A	0.367A	1098.2A	1600.4A
	Control	271.31C	35.44B	0.417B	890.1B	1181.6B

*Means in the same column of each host plant with the same letter are not significantly different at *P* = 0.05.*

**FIGURE 4:**
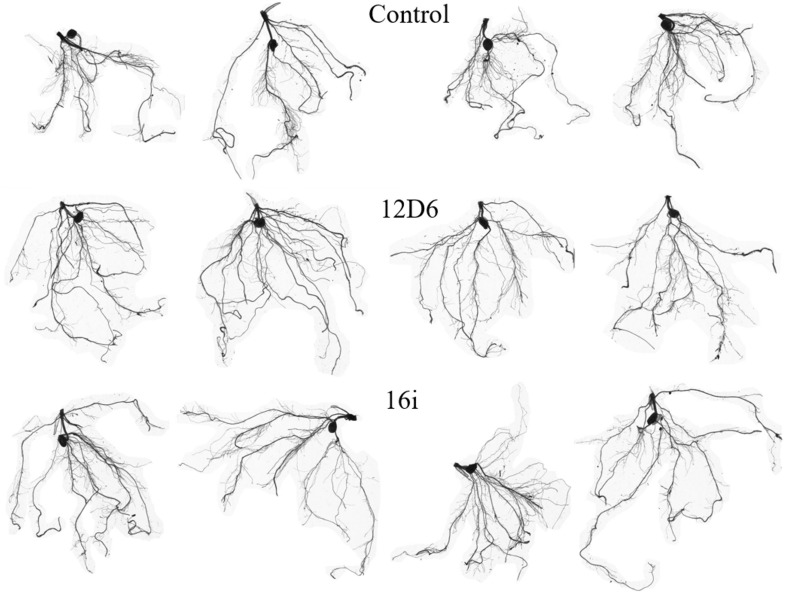
Root system architecture in maize seedlings treated with the control **(top)**, *Bacillus* sp. (12D6) **(center)** and *Enterobacter* sp. (16i) **(bottom)** after exposure to 7 days of continuous water deficit.

In maize, the root systems of seedlings (Figure 4) treated with either bacterial inoculum were larger in terms of total root length and surface area and had more root tips than non-inoculated seedlings (Table 2). Some differences between the treatments in other metrics were observed. The seedlings treated with *Enterobacter* sp. (16i) had longer total root length, more branching and smaller average root diameter compared with those treated with *Bacillus* sp. (12D6) or the controls (Table 2).

Targeted analyte LC/MS based phytohormone profiling of PGPR strains grown *in vitro* revealed that both strains produced indole-3-acetic acid (IAA) and salicylic acid (SA) (Supplementary Figure S1) in relatively high amounts (*P* < 0.0005) compared to the other phytohormones profiled and the LB control (*P* < 0.0001). The analytes were found both in the pelleted cells and the filtrate compared to the LB control, indicating both PGPR strains may secrete both compounds.

## Discussion

This study reports the development and use of a bioprospecting pipeline to effectively screen PGPR for the ability to rapidly mitigate plant drought stress symptoms in multiple cereal hosts when applied to plants at the onset of water deficit conditions. By starting with samples of perennial grasses (bermudagrass) that appeared healthy under constant water deficit conditions in the semi-arid environment of El Paso, TX, we attempted to focus on rhizosphere microbiomes that may be selected for and adapted to mitigating drought tolerance to grasses under these conditions. The pre-screening approach was based on selection of PGPR that mediated the desired seedling phenotype of delayed onset and severity of drought symptoms. This screening procedure succeeded in selecting specific PGPR capable of producing these results rapidly and under water stress conditions.

Using this pipeline, two PGPR strains were identified as *Bacillus* sp. (12D6) and *Enterobacter* sp. (16i). Both wheat and maize seedlings experienced a delay in the onset of drought symptoms when treated with either isolate, although visual assessment of plant performance suggested strain 12D6 was somewhat more effective in mitigating drought symptoms in wheat, whereas strain 16i was more effective in maize. These phenotypic differences in seedling tolerance of drought stress were associated with changes in root system architecture, although there were some differences between hosts in response to the PGRP strains. For instance, in wheat, although both strains had a significant effect on root system architecture, producing more branched roots than non-inoculated seedlings, 12D6-treated seedlings also produced larger root systems in terms of total root length than 16i-treated seedlings or the controls. In maize, both strains produced larger root systems in terms of total root length and surface area and had more root tips, compared with non-inoculated seedlings. However, the root systems of 16i-treated seedlings also had greater total root length, more branches, and smaller average root diameters than those of 12D6-treated seedlings or the controls.

The production of greater linear root length, surface area, and more root tips has been correlated previously with better water stress tolerance and overall improvements in maintaining plant productivity under drought (Comas et al., 2013). Root system length and surface area contribute to better soil exploration, whereas the proliferation of higher order roots resulting in more root tips are important for root water uptake capacity (Vardharajula et al., 2011; Naseem and Bano, 2014; Ngumbi and Kloepper, 2016; Barnawal et al., 2017). Previous research demonstrates that reductions in root diameter may enable faster relative growth rates and rapid resource acquisition through expansion of the root system coupled with lower investment in dry biomass (Garnier, 1992; Wahl and Ryser, 2000; Birouste et al., 2014). Although hosts differed somewhat in how their root systems responded to PGPR treatment, in general these results suggest that water stress tolerance resulted in part from bacterially mediated changes in root system architecture that may have led to enhanced avoidance of drought stress symptoms.

Previous research suggests that host-specific selection of and response to PGPR are complex (Kloepper, 1996; Smith and Goodman, 1999; Drogue et al., 2012). For example, differences in the response of spring wheat to *Bacillus* sp. at the cultivar level have been observed (Chanway et al., 1988). At the molecular level, plant-microbe specificity may be driven by plant and microbial signals important for host-microbe perception, microbial recruitment, and microbial initiation of host response to symbiosis traits (Smith and Goodman, 1999). In the case of drought tolerance-mitigating PGPR, bacterial adaptation to water stress (e.g., EPS production), and host specific responses to drought stress (e.g., root system architecture, stomatal closure) also may be important. Success in mediating water stress tolerance by PGPR ultimately depends on effective root colonization, reliable expression of microbial traits important for PGPR activity, and cultivar specific differences in mechanisms of adaptation to drought stress (Kloepper, 1996; Drogue et al., 2012). Although both strains successfully colonized the rhizosphere at concentrations of at least 10^6^ CFU g^–1^ sample (root and rhizosphere soil), any of these other factors may have contributed to the observed differences in the effectiveness 12D6 and 16i in mitigating drought stress in maize and wheat.

Production and secretion of bacterial compounds that may serve as stimulators of plant growth and development or signals within whole-plant signaling pathways (e.g., phytohormones) have been reported to be involved in bacterially mediated drought tolerance in plants (Dodd et al., 2010; Bakker et al., 2014; Ngumbi and Kloepper, 2016). Our LC-MS phytohormone profiling of bacterially produced compounds demonstrated that both 12D6 and 16i bacterial strains produced IAA and SA in cellular components and supernatant fractions when grown in LB liquid overnight (Supplementary Figure S1).

Bacteria have multiple pathways for IAA biosynthesis, which may function in tryptophan storage, and regulation of tryptophan-dependent IAA biosynthesis may have wide-spread effects on bacterial gene expression patterns (Spaepen et al., 2007; Spaepen and Vanderleyden, 2011; Duca et al., 2014). Research has shown that that bacterially produced IAA may function in microbe–microbe signaling and is important for establishing symbiotic relationships with plants, such as during nodule or tumor formation (Spaepen et al., 2007). It is presumed that over 80% of all bacteria isolated from the rhizosphere can produce IAA (Patten and Glick, 1996; Duca et al., 2014). In plants, endogenously produced IAA serves as a phytohormone involved in the regulation of plant growth and development, including the root system. Exogenous application of IAA causes alterations in root system architecture that appear to depend on IAA concentration. For example, low concentrations of IAA generally stimulate primary root elongation, whereas high IAA levels may diminish primary root growth and stimulate the formation of lateral roots and root hairs (Patten and Glick, 2002; Vacheron et al., 2013). The application of IAA-producing PGPR has been shown to produce similar root system responses, which have been linked to plant drought stress tolerance (Marulanda et al., 2009; Bresson et al., 2013; Ngumbi and Kloepper, 2016). Moreover, the specific role of IAA in mediating these phenotypes was demonstrated via comparison of growth promoting activity by auxin-producing PGPR and auxin-deficient mutants (Patten and Glick, 2002; Vacheron et al., 2013). For example, canola seedlings treated with the auxin-producing PGPR *Pseudomonas putida* GR12-2 produced longer roots compared to seedlings treated with an auxin-deficient mutant or the untreated control. Cell-free supernatants of the wild type also enhanced the proliferation of adventitious roots on mung bean cuttings compared to supernatants of the mutant or the control (Patten and Glick, 2002; Vacheron et al., 2013). In contrast, bacterial production of IAA at high concentrations may have inhibitory on root growth and elongation, as demonstrated by the application of IAA overexpression derivatives (Sarwar and Kremer, 1995; Xie et al., 1996). In the present study, the alterations in root system architecture of both wheat and maize seedlings associated with the application of either strain are consistent with the hypothesis that bacterially produced IAA may have contributed to these phenotypes, and this hypothesis merits further investigation.

Production of SA among rhizosphere-colonizing bacteria has been shown to be widespread and some strains can produce significant amounts when cultivated *in vitro*. For example, there are reported cases of *Pseudomonas fluorescens* biocontrol SA “super-producers” that can synthesize concentrations of SA up to 55 μg per ml *in vitro* (Bakker et al., 2014). SA production may be significantly increased under water stress, as observed for PGPR strains *Achromobacter xylosoxidans*, *B. pumilus* SF3, and *B. pumilus* SF4 (Forchetti et al., 2010). In plants, endogenously produced SA serves as a phytohormone involved in stress response. Although primarily studied for its involvement in activating systemic acquired resistance SAR in defense of biotic stresses, SA has also been shown to aid in abiotic stress tolerance, including drought (Wituszynska et al., 2013; Khan et al., 2018). Both phytohormones SA and abscisic acid (ABA) have been proposed to increase drought tolerance through the accumulation of induced ROS and induced signaling of stomatal closure (Daszkowska-Golec and Szarejko, 2013). By eliciting stomatal closure, these phytohormones can reduce transpirational water loss and allow for increasing water storage in the above ground tissue during drought conditions. It is therefore intriguing to speculate that bacterial production of SA may be involved in abiotic stress tolerance via its contribution to the endogenously produced plant SA pools and SA signaling pathways. However, despite the numerous examples of PGPR that produce SA and induce biotic or abiotic stress tolerance, there is very little evidence for the direct role of bacterially produced SA in these processes (Bakker et al., 2014). As Bakker et al. (2014) argue in a 2014 review of rhizobacterial salicylate production, although many root-inhabiting bacteria produce SA *in vitro*, in the rhizosphere they most likely excrete SA primarily as SA-based siderophores under iron limiting conditions or as an adaptation to high temperature conditions when other siderophore molecules are no longer functioning. In contrast to the lack of effect on plants, bacterially produced SA has been shown to be involved in the regulation of key bacterial traits necessary for rhizosphere survival and thus may be important for regulating bacterial community dynamics under drought stress conditions (Bakker et al., 2014). The production of SA by both strains selected for root colonization under drought stress conditions via our bioprospecting pipeline would seem to support this hypothesis.

In summary, the development and application of a novel bioprospecting pipeline effectively screened PGPR for the capacity to rapidly mitigate seedling drought stress symptoms. The screen isolated and identified two PGPR candidates of *Bacillus* sp. (12D6) and *Enterobacter* sp. (16i). Compared to untreated controls, both wheat and maize seedlings treated with either strain were significantly more vigorous following a 7-day water deficit and displayed alterations in root system architecture that likely facilitated the drought avoidance phenotype. The ability of both strains to survive and rapidly protect both wheat and maize seedlings when applied at the onset of drought is a positive indicator of their potential for mitigating seedling drought stress in cereal cropping systems, which will be tested in future research.

## Data Availability

The data that support the findings in this study are available from the corresponding author upon reasonable request.

## Author Contributions

MJ conceived the study, prepared the figures, and wrote the manuscript. GN collected the samples. MJ, EB, and KM performed laboratory assays and data analysis. Y-KJ, MK, and EP conceived the study and contributed resources. All authors edited the manuscript and approved the final draft.

## Conflict of Interest Statement

The authors declare that the research was conducted in the absence of any commercial or financial relationships that could be construed as a potential conflict of interest.
