# Supplementary figures and images for: Bioprospecting Plant Growth-Promoting Rhizobacteria That Mitigate Drought Stress in Grasses
Source: Front Microbiol. 2019 Sep 10;10:2106. doi: 10.3389/fmicb.2019.02106 (PMC6747002; doi:10.3389/fmicb.2019.02106)

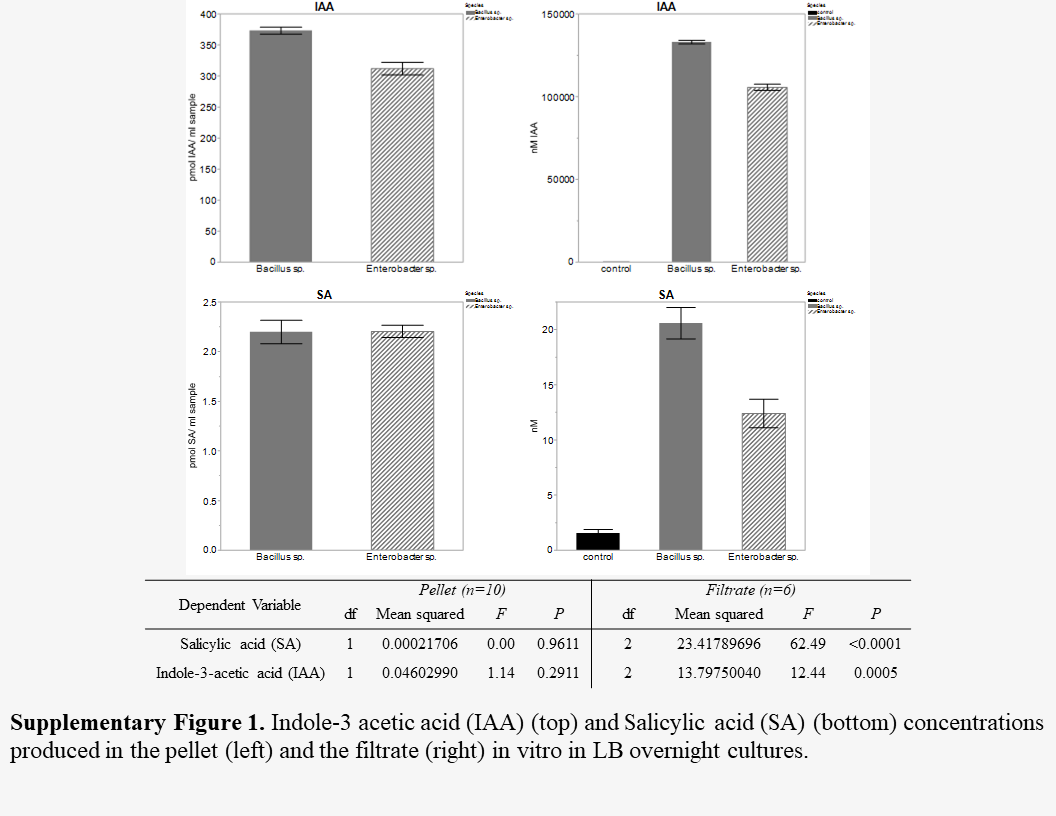

Supplement: Supplementary file 1 [file Image_1.TIF]
